# 5-Lipoxygenase Deficiency Reduces Acetaminophen-Induced Hepatotoxicity and Lethality

**DOI:** 10.1155/2013/627046

**Published:** 2013-10-31

**Authors:** Miriam S. N. Hohmann, Renato D. R. Cardoso, Felipe A. Pinho-Ribeiro, Jefferson Crespigio, Thiago M. Cunha, José C. Alves-Filho, Rosiane V. da Silva, Phileno Pinge-Filho, Sergio H. Ferreira, Fernando Q. Cunha, Rubia Casagrande, Waldiceu A. Verri

**Affiliations:** ^1^Department of Pathology, Biological Science Centre, State University of Londrina, Rodovia Celso Garcia Cid Pr 445, Km 380. Cx. Postal 6001, 86051-990 Londrina PR, Brazil; ^2^Department of Pharmacology, Ribeirão Preto Medical School, University of São Paulo, Avenida Bandeirantes 3900, 14049-900 Ribeirão Preto, SP, Brazil; ^3^Department of Pharmaceutical Sciences, Health Sciences Centre, State University of Londrina, Rodovia Celso Garcia Cid Pr 445, Km 380, Cx. Postal 10011, 86051-990 Londrina, PR, Brazil

## Abstract

5-Lipoxygenase (5-LO) converts arachidonic acid into leukotrienes (LTs) and is involved in inflammation. At present, the participation of 5-LO in acetaminophen (APAP)-induced hepatotoxicity and liver damage has not been addressed. 5-LO deficient (5-LO^−/−^) mice and background wild type mice were challenged with APAP (0.3–6 g/kg) or saline. The lethality, liver damage, neutrophil and macrophage recruitment, LTB_4_, cytokine production, and oxidative stress were assessed. APAP induced a dose-dependent mortality, and the dose of 3 g/kg was selected for next experiments. APAP induced LTB_4_ production in the liver, the primary target organ in APAP toxicity. Histopathological analysis revealed that 5-LO^−/−^ mice presented reduced APAP-induced liver necrosis and inflammation compared with WT mice. APAP-induced lethality, increase of plasma levels of aspartate aminotransferase and alanine aminotransferase, liver cytokine (IL-1**β**, TNF-**α**, IFN-**γ**, and IL-10), superoxide anion, and thiobarbituric acid reactive substances production, myeloperoxidase and N-acetyl-**β**-D-glucosaminidase activity, Nrf2 and gp91^phox^ mRNA expression, and decrease of reduced glutathione and antioxidant capacity measured by 2,2′-azinobis(3-ethylbenzothiazoline 6-sulfonate) assay were prevented in 5-LO^−/−^ mice compared to WT mice. Therefore, 5-LO deficiency resulted in reduced mortality due to reduced liver inflammatory and oxidative damage, suggesting 5-LO is a promising target to reduce APAP-induced lethality and liver inflammatory/oxidative damage.

## 1. Introduction 

The 5-lipoxygenase (5-LO) pathway converts arachidonic acid into leukotrienes (LTs), specifically LTB_4_ and cysteinyl-LTs (LTC_4_, LTD_4_, and LTE_4_) [1], and studies indicate that this pathway is responsible for developing and sustaining inflammation [2]. All LTs display a variety of proinflammatory actions, but LTB_4_, in particular, is one of the most potent chemotactic agents and activating factors for leukocytes [3]. In fact, LTB_4_ has been implicated in the pathophysiology of acute and chronic inflammatory diseases [4, 5].

In this context, many studies have investigated the participation of 5-LO and its products in different experimental models of liver injury. 5-LO pathway and LTs have been implicated in hepatic inflammation and liver damage. Elevated production of LTs in the liver was detected in rats with CCl_4_-induced liver injury [6, 7]. In hepatic ischemia and reperfusion injury, LT production was enhanced and associated with the development of hepatic edema and dysfunction [8]. Furthermore, LTB_4_ and the 5-LO pathway were reported to be involved in the pathogenesis of experimental liver fibrosis and inflammatory necrosis [7, 9, 10]. Thus, 5-LO products are important mediators of hepatic inflammation and cell injury.

Considering that LTs are important mediators of liver inflammation and damage, 5-LO pathway and its products might also be involved in acetaminophen (APAP)-induced liver injury. APAP is a widely used over-the-counter analgesic and antipyretic with few side effects when taken at therapeutic doses. However, APAP intoxication can result in severe liver damage characterized by centrilobular liver necrosis and, in more severe cases, acute liver failure and eventually death [11]. Studies have shown that APAP overdose has become the most common cause of acute liver failure in many Western countries [12, 13].

It is well established that APAP-induced liver injury depends on the metabolic conversion of APAP to the highly reactive metabolite *N-*acetyl-*p*-benzoquinone imine (NAPQI) by cytochrome P-450 (CYP) enzymes, primarily CYP2E1 and to a lesser extent CYP1A2, CYP2A6, and CYP3A4 [14, 15]. In normal conditions, this metabolite is readily detoxified by hepatic reduced glutathione (GSH). However, after toxic dose of APAP, GSH is depleted, and, as a result, NAPQI accumulates and covalently binds to hepatocellular proteins, initiating liver injury [16, 17]. Although protein binding is the initiator of toxicity and cellular injury, secondary processes amplify and propagate it. Initial hepatocellular damage caused by NAPQI can lead to the release of damage-associated molecular patterns (DAMPS) such as high-mobility group box 1 protein (HMGB1), heat shock proteins, DNA fragments, and others [18], which can directly activate nonparenchymal hepatic cells, Kupffer cells (KC), and other innate immune cells [19, 20]. In addition, these cells can also be activated by potent chemotactic factors (i.e., LTB_4_) released by injured hepatocytes [21]. Activated cells can release a wide range of inflammatory mediators, such as reactive oxygen species (ROS) and reactive nitrogen species (RNS) [22], proinflammatory cytokines [18], and LTs [6] amplifying the oxidative stress and inflammatory responses, consequently promoting further hepatic injury [23]. Moreover, the excessive ROS and RNS production during inflammation contributes to the depletion of GSH [24], which could result in further NAPQI accumulation [17].

Neutrophil and macrophage accumulation in the liver has also been associated with APAP-induced liver injury [25, 26] and several other experimental animal models of liver injury such as ischemia-reperfusion injury [27, 28] and endotoxemia [29]. Neutrophils and macrophages under certain circumstances can severely aggravate tissue damage. In APAP-induced hepatotoxicity, excessive neutrophil and macrophage activity can contribute to liver inflammation and injury [25, 26].

Although many studies have demonstrated that 5-LO pathway is involved in the pathogenesis of different models of liver inflammation, at present the participation of 5-LO in APAP-induced hepatotoxicity and liver damage has not been addressed. In this context, the aim of the current study was to use 5-LO deficient mice to investigate the role of 5-LO on APAP-induced lethality and liver damage. In addition, it aimed to demonstrate the mechanisms by which 5-LO deficiency ameliorates the events mentioned previously focusing on neutrophil and macrophage recruitment, cytokine production, and oxidative stress in the liver.

## 2. Methods 

### 2.1. Animals

Male 5-lipoxygenase deficient (5-LO^−/−^) and wild type (WT, SV129) mice weighing 20–25 g were used. 5-LO^−/−^ and WT mice, originally from The Jackson Laboratory (Bar Harbor, ME, USA), were gifted from Dr. Marc Lee Peters-Golden (University of Michigan) weighing 20–25 g. Mice were housed at 21°C on a 12 h light/dark cycle in standard clear plastic cages with food and water *ad libitum.* Animals were handled humanely, and all procedures concerning animal care and use were approved by the Research and Ethics Committee of State University of Londrina (process 2961/2010-10) and carried out in accordance with its guidelines.

### 2.2. Experimental Design

In dose-response experiments, WT mice (*n* = 10 per group) were treated orally with 0.3, 1, 2, 3, and 6 g/kg of APAP suspended in saline (200 mg/mL). In APAP-induced lethality experiments, 5-LO^−/−^ and WT mice (*n* = 10 per group) were treated orally with 3 g/kg of APAP suspended in saline (200 mg/mL of saline) or equal volume of saline (control group). In these survival experiments, mice were observed every 6 h during 72 h, and any that showed extreme distress or became moribund were sacrificed. For subsequent experiments, animals were anesthetized and sacrificed 12 h after APAP treatment. Blood samples were collected by cardiac puncture to determine the enzymatic activities of aspartate aminotransferase (AST) and alanine aminotransferase (ALT). For liver analysis, the abdomen of the mice was opened, and the left lobe of the liver was quickly excised. A portion of the tissue was stored at −20°C in 50 mM K_2_HPO_4_ buffer (pH 6.0) containing 0.5% hexadecyl trimethylammonium bromide (HTAB) for myeloperoxidase (MPO) and N-acetyl-*β*-D-glucosaminidase (NAG) activity determination, and the remaining tissue was stored in saline at −80°C for LTB_4_ and cytokine (TNF-*α*, IL-1*β*, IFN-*γ*, and IL-10) quantification. The midsections of the left lobe of the liver were collected and processed for histopathology analysis. In order to assess hepatic oxidative stress, the liver was first perfused with 10 mL of ice-cold 1.15% KCl solution through the portal vein. For quantitative PCR (qPCR) analysis, the 1.15% KCl solution used for liver perfusion was treated with 0.1% v/v diethylpyrocarbonate (DEPC) for 24 hours at 37°C and then autoclaved to inactivate DEPC. The left lobe was excised in the same manner, and a portion of the perfused tissue was separated for 2,2′-azinobis-(3-ethylbenzothiazoline-6-sulfonate; ABTS) assay, whereas the remaining tissue was immediately stored at −80°C for later assessment of reduced glutathione (GSH), thiobarbituric acid reactive substances (TBARS) levels, nitroblue tetrazolium (NBT) reduction, and mRNA expression for gp91^phox^ and transcription factor Nrf2.

### 2.3. Enzymatic Markers of Liver Injury

Blood was collected into microtubes containing 50 *μ*L of the anticoagulant ethylenediamine tetraacetic acid (EDTA) (5,000 IU/mL) and centrifuged (200 ×*g*, 10 min, 4°C), and the plasma was separated. In order to determine enzymatic activities of AST and ALT, plasma samples were processed according to the manufacturer's instructions (Labtest Diagnóstico S.A., Brazil). Results were presented as U/L.

### 2.4. Histopathology

For liver histopathology analysis, midsections of the left lobes of the liver were fixed in 10% buffered neutral formalin solution for 24 h and embedded in paraffin wax, and 5 *µ*m sections were prepared and stained with hematoxylin—eosin (H&E). H&E stained liver sections were examined and scored by a pathologist using light microscopy, and the degree of necrosis and inflammation was determined as previously described by Yaman et al. [30]. The degree of necrosis was classified on a scale of 0–3 [normal: 0 (0%), mild: 1 (1–25%), moderate: 2 (26–49%), severe: 3 (50–100%)] and expressed as the mean of 10 high power fields (HPFs), chosen at random. The degree of inflammation was evaluated in the same 10 HPFs and classified on a scale of 0–3 [no inflammation:  0 (mean of inflammatory cells in 10 HPFs = 0), weak inflammation:  1 (mean of inflammatory cells in 10 HPFs = 1–10), moderate inflammation: 2 (mean of inflammatory cells in 10 HPFs = 11–49), and severe inflammation: 3 (mean of inflammatory cells in 10 HPFs = 50 and over)].

### 2.5. Cytokines and LTB_4_ Levels

Frozen liver samples were homogenized in 500 *μ*L of saline using a turrax T10 basic (IKA, Staufen, Germany) in an ice bath. The samples were centrifuged (800 ×*g*, 10 min, 4°C), and with the resulting supernatant, IL-1*β*, TNF-*α*, IFN-*γ*, IL-10 (eBioscience), and LTB_4_ (Cayman Chemical, Ann Arbor, MI, USA) levels were determined by ELISA, according to the manufacturer's instructions. Results were presented as pg/mg of liver.

### 2.6. MPO Activity

MPO is an enzyme abundantly present in the azurophilic granules of neutrophils that has been used as a biochemical marker of neutrophil infiltration into various tissues [24, 31]; thus in the present study MPO colorimetric assay was used to assess neutrophil migration to the liver. Frozen samples were homogenized as described above and centrifuged (16,100 ×*g*, 2 min, 4°C). The resulting supernatant was assayed spectrophotometrically for MPO activity determination at 450 nm. Briefly, 5 *μ*L of the supernatant was mixed with 200 *μ*L 50 mM phosphate buffer (pH 6.0), containing 0.167 mg/mL *o*-dianisidine dihydrochloride and 0.015% hydrogen peroxide. The results were presented as the MPO activity (U/mg of liver) [24, 31].

### 2.7. NAG Activity

NAG activity was determined by an adapted colorimetric method previously described by Horinouchi et al. [32]. Briefly, 20 *μ*L of supernatant, previously described in MPO activity, was placed in a 96-well plate, followed by the addition of 80 *μ*L of 50 mM phosphate buffer, pH 6.0. The reaction was initiated by the addition of 2.24 mM 4-nitrophenyl N-acetyl-*β*-D-glucosaminide. The plate was incubated at 37°C for 10 min, and the reaction was stopped by the addition of 100 *µ*L of 0.2 M glycine buffer, pH 10.6. The enzymatic activity was determined spectrophotometrically at 400 nm (Multiskan GO Microplate Spectrophotometer, ThermoScientific, Vantaa, Finland). NAG activity of samples was presented as NAG activity (OD/mg of liver). 

### 2.8. GSH

GSH levels were determined spectrophotometrically by an adapted method described by Sedlack and Lindsay [33]. The frozen perfused liver samples were homogenized in cold 0.02 M EDTA. The homogenate was treated with 50% trichloroacetic acid and centrifuged (1,500 ×*g*, 15 min), and to the supernatant 0.4 M Tris-HCl, pH 8.9 was added. Next, samples were vortex-mixed, and 10 mM dithiobisnitrobenzoic acid was added, followed by vortex-mixing. Samples were allowed to stand for 5 minutes before being read at 412 nm. Standard curve was prepared using different concentrations of GSH, in addition to the other reagents mentioned before. Results were presented as nmol GSH/mg of liver.

### 2.9. ABTS Assay

The perfused liver samples collected were immediately processed, and homogenates (20% w/v) were prepared with ice-cold 1.15% KCl. Samples were centrifuged (200 ×*g*, 10 min, 4°C), and with the resulting supernatant total antioxidant capacity of liver was assessed by ABTS assay [34]. This assay is based on the ability of the antioxidants molecules to quench ABTS radical cation (ABTS^*·*+^), a blue-green chromophore with characteristic absorption at 734 nm, compared with that of Trolox. The antioxidants present in the liver samples when added to ABTS^*·*+^ reduce it into ABTS, which results in decolorization. ABTS^*·*+^ was produced by reacting ABTS stock solution (ABTS dissolved in water to a 7 mM concentration) with 2.45 mM potassium persulfate (final concentration) and allowing the mixture to stand in the dark at room temperature for 12–16 h before use. For the study, ABTS^*·*+^ solution was diluted in phosphate buffer pH 7.4 to reach an absorbance of 0.8 (±0.02) at 734 nm. 10 *μ*L of the samples was added to 1 mL of the diluted ABTS^*·*+^ solution; samples were vortex-mixed and allowed to stand for 6 min. The samples were read in spectrophotometer at 734 nm. A standard curve was prepared using different concentrations of Trolox. Because this is a Trolox equivalent antioxidant capacity (TEAC) assay, results were presented as *µ*mol Trolox equivalent/mg of liver.

### 2.10. NBT Reduction

The superoxide anion production was determined by the reduction of the redox dye NBT [35]. Frozen liver samples were homogenized with 500 *μ*L of saline, and 50 *μ*L of the homogenate was placed in a 96-well plate, followed by the addition of 100 *μ*l of NBT solution (1 mg/mL) and incubation for 1 h at 37°C. The supernatant was carefully removed, and the formazan precipitated was then solubilized by adding 120 *μ*L of 2 M KOH and 140 *μ*L of DMSO. The optical density was measured by microplate spectrophotometer reader (Multiskan GO Microplate Spectrophotometer, ThermoScientific, Vantaa, Finland) at 600 nm. The weight of samples was used for data normalization, and results were presented as NBT reduction (OD/mg of liver).

### 2.11. Lipid Peroxidation

Lipid peroxidation in the liver was assessed by determining TBARS levels using an adapted method previously described by Guedes et al. [36]. For this assay, trichloroacetic acid (10%) was added to the homogenate to precipitate proteins followed by centrifugation (1,000 ×*g*, 3 min, 4°C). The protein-free supernatant was separated, and thiobarbituric acid (0.67%) was added. The mixture was kept in water bath (15 min, 100°C). Malondialdehyde (MDA), an intermediate product of lipid peroxidation, was determined by difference between absorbances at 535 and 572 nm using a microplate spectrophotometer reader. The results were presented as TBARS (nmol MDA/mg of liver).

### 2.12. Quantitative Polymerase Chain Reaction (qPCR)

qPCR was performed as previously described [37]. Samples were homogenized in TRIzol reagent, and total RNA was extracted by using the SV Total RNA Isolation System (Promega). All reactions were performed in triplicate using the following cycling conditions: 50°C for 2 min, 95°C for 2 min, followed by 40 cycles of 95°C for 15 s and 60°C for 30 s. qPCR was performed in a LightCycler Nano Instrument (Roche, Mississauga, ON, USA) sequence detection system by using the Platinum SYBR Green qPCR SuperMix UDG (Invitrogen, USA). The primers used were gp91^phox^, sense: 5′-AGCTATGAGGTGGTGATGTTAGTGG-3′, antisense: 5′-CACAATATTTGTACCAGACAGACTTGAG-3′; Nrf2, sense: 5′-TCACACGAGATGAGCTTAGGGCAA, antisense: 5′-TACAGTTCTGGGCGGCGACTTTAT, *β*-actin, sense: 5′-AGCTGCGTTTTACACCCTTT-3′, antisense: 5′-AAGCCATGCCAATGTTGTCT-3′. The expression of *β*-actin mRNA was used as a control for tissue integrity in all samples.

### 2.13. Sleeping Time Induced by Pentobarbital 

APAP-induced-toxicity is highly dependent on metabolic conversion of APAP to NAPQI by CYP enzymes; therefore the effect of 5-LO deficiency on hepatic microsomal cytochrome P450 activity was investigated by assessing pentobarbital-induced sleeping time in 5-LO^−/−^ and WT mice. 5-LO^−/−^ and WT mice were treated with pentobarbital diluted in saline (50 mg/kg, i.p.), and the duration of sleep (min) of each animal was analyzed. Loss of righting reflex to recovery was recorded as the sleeping time [38].

### 2.14. Statistical Analysis

The results are expressed as mean ± S.E.M. Survival rates were estimated by the Kaplan-Meier method, and statistical analysis was carried out by the log-rank test to test for equality of the survival curves. Statistical differences were compared by Student's *t*-test or by one-way ANOVA followed by Bonferroni's multiple comparison test. For categorical variables, the Kruskal-Wallis test followed by Dunn's test was performed. All statistical analyses were performed using Graph Pad Prism 5 (La Jolla, CA). The level of significance was set at *P* < 0.05. Studies were conducted two to three times, and mean data are shown.

## 3. Results 

### 3.1. APAP Induces Dose-Dependent Lethality and LTB_4_ Production in the Liver

**Figure 1:**
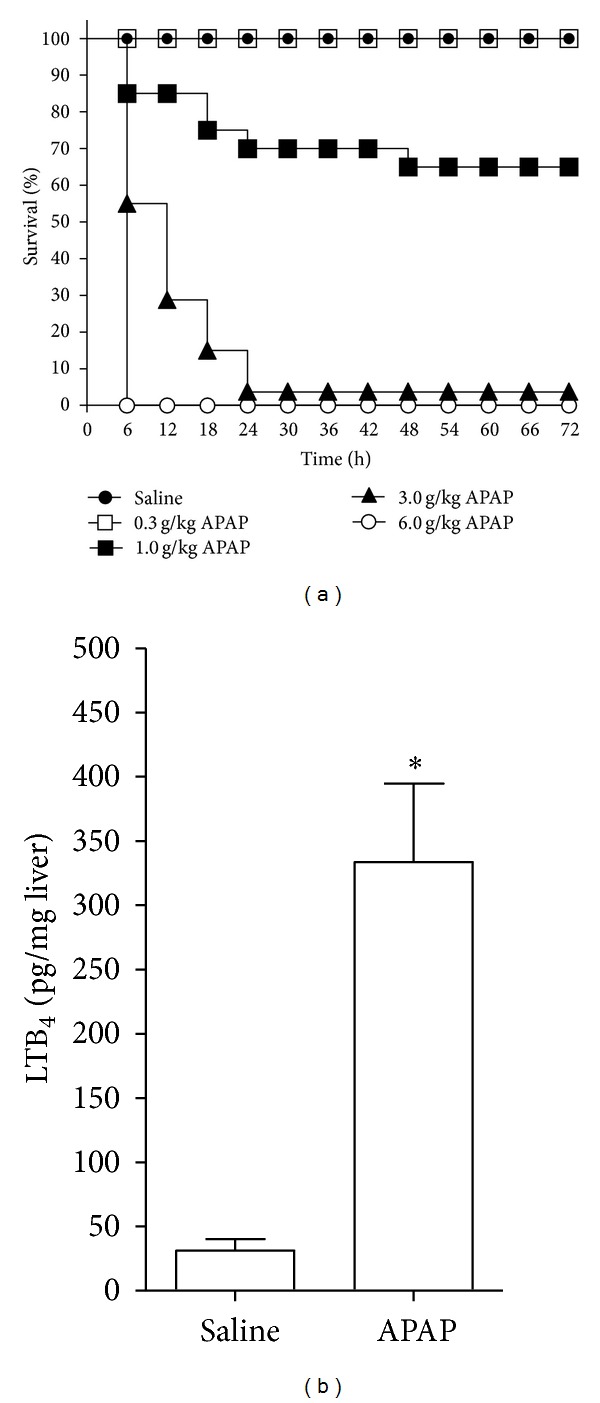
Acetaminophen (APAP) induces dose-dependent lethality and LTB_4 _ production in the liver. (a) WT mice were treated with APAP (0.3, 1, 3, and 6 g/kg) or saline per oral, and lethality was assessed. The lethality induced by APAP was monitored at 6 h intervals during 72 h. *n* = 10, representative of three separate experiments. (b) WT mice were treated with APAP (3 g/kg, per oral) or saline, and after 12 h liver samples were collected for the determination of LTB_4 _ levels by ELISA. Values are mean ± S.E.M., *n* = 5, representative of two separate experiments. *P < 0.05 compared to saline group, Student's *t*-test.

To determine the dose of APAP necessary to induce significant lethality in this strain, a dose-response study was performed. WT mice were treated orally with APAP (0.3, 1, 2, 3, and 6 g/kg) or equal volume of saline, and lethality was assessed. Saline and 0.3 g/kg of APAP did not induce death in any of the animals (Figure 1(a)). The administration of 1.0 g/kg of APAP induced 15% lethality in 6 h, 25% in 18 h, 30% in 24 h, and 35% in 48 h, which was maintained until the end of the experiment. The administration of 2 g/kg induced similar lethality, 20% in 12 h and 35% in 66 h, which was also maintained. Mice treated with 3 g/kg of APAP presented 45% and 70% mortality within 6 h and 12 h, respectively, and a little over 95% in 24 h, which was maintained. Finally, mice were treated with 6 g/kg of APAP to assure that 3 g/kg of APAP was the submaximal lethal dose in this experimental model. The administration of 6 g/kg of APAP induced 100% mortality in 6 h, thus considered inadequate (Figure 1(a)). Therefore, 3 g/kg of APAP was selected for the following experiments addressing the hepatic mechanisms triggered by a lethal dose of APAP.

In order to determine 5-LO participation in APAP hepatotoxicity, the effect of the toxic dose of APAP on hepatic levels of LTB_4 _ was assessed. In this context, WT mice received 3 g/kg of APAP or equal volume of saline per oral, and after 12 h animals were sacrificed and liver samples were collected for assessment of LTB_4 _ levels. It was observed that APAP induced a ~10-fold increase of LTB_4_ levels in the liver compared to saline (Figure 1(b)). Twelve h was selected since it is an intermediary time point between intoxication and death (Figure 1(b)).

### 3.2. 5-LO Participates in APAP-Induced Lethality

**Figure 2:**
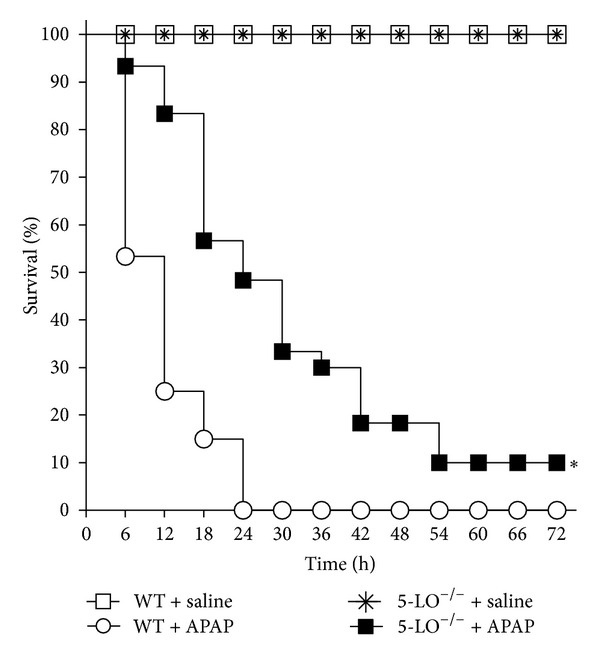
5-LO participates in acetaminophen (APAP)-induced lethality. WT mice and 5-LO^−/−^ mice were treated with APAP (3 g/kg) or saline per oral. The lethality induced by APAP was monitored at 6 h intervals during 72 h. *n* = 10, representative of three separate experiments. *P < 0.001 compared to WT mice treated with APAP, Kaplan-Meier method, followed by the log-rank test.

5-LO^−/−^ and WT mice were treated with APAP (3 g/kg) or equal volume of saline per oral, and survival rates during APAP intoxication were determined (Figure 2). APAP administration induced significant mortality in WT mice, with approximately 45% lethality in 6 h, 75% in 12 h, and 100% in 24 h. However, APAP induced significantly lower mortality in 5-LO^−/−^ mice compared to WT mice: 5% lethality in 6 h, 15% in 12 h, 60% in 24 h, and 90% in 54 h in 5-LO^−/−^ mice. Saline did not induce death in any of the animals. Twelve h was selected for the next experiments investigating the mechanisms involved in APAP-induced intoxication because the greatest difference between WT and 5-LO^−/−^ was observed at this time point.

### 3.3. APAP-Induced Histopathological Changes in the Liver Were Reduced in 5-LO^−/−^Mice

**Figure 3:**
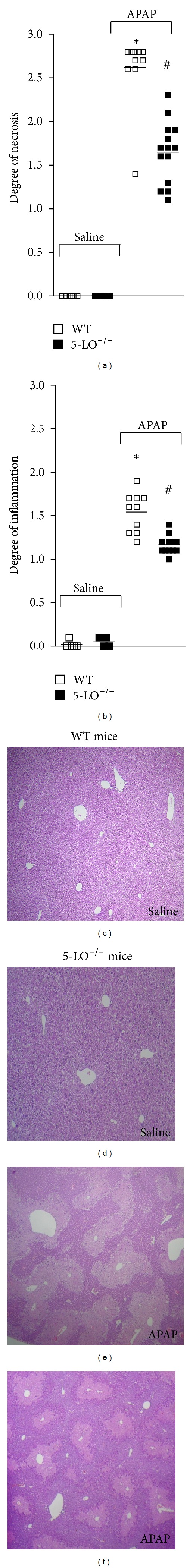
Acetaminophen (APAP) induces 5-LO-dependent histopathological changes in the liver. WT and 5-LO^−/−^ mice were treated with APAP (3 g/kg) or saline per oral, and after 12 h liver samples were collected and processed for histopathology analysis. The degree of liver necrosis (a) (*n* = 5 for saline groups, *n* = 11 for WT APAP group, and *n* = 14 for 5-LO^−/−^ APAP group) and inflammation (b) (*n* = 4-5 for saline groups, *n* = 10 for WT APAP group, and *n* = 2 for 5-LO^−/−^ APAP group) were assessed. *P < 0.05 compared to saline-treated WT and 5-LO^−/−^ mice and ^#^ *P* < 0.05 compared to APAP-treated WT mice. Kruskal-Wallis test was followed by Dunn's multiple comparison test. (c–f) Representative images of histopathological changes in the liver (H&E 40×). (c) WT mice treated with saline, (d) 5-LO^−/−^ mice treated with saline, (e) WT mice treated with APAP, and (f) 5-LO^−/−^ mice treated with APAP.

APAP (3 g/kg) or equal volume of saline per oral was administrated to 5-LO^−/−^ and WT mice, and after 12 h liver histopathological analysis was performed and representative images of liver histology were obtained. Histopathology analysis of the liver demonstrated that APAP induced significantly higher degree of liver necrosis (Figure 3(a)) and inflammation (Figure 3(b)) in WT mice when compared to 5-LO^−/−^ mice. Saline did not induce necrosis (Figure 3(a)) or inflammation (Figure 3(b)) in the liver of WT and 5-LO^−/−^ mice. In representative images of liver histology, it can be observed that APAP induced significantly larger area of necrosis in the liver of 5-LO^−/−^ mice when compared to WT mice (Figures 3(e) and 3(f), resp.). No apparent difference was observed in the liver samples of WT and 5-LO^−/−^ mice receiving only saline (Figures 3(c) and 3(d)).

### 3.4. APAP-Induced Increase in Plasmatic AST and ALT Levels Was Reduced in 5-LO^−/−^ Mice

**Figure 4:**
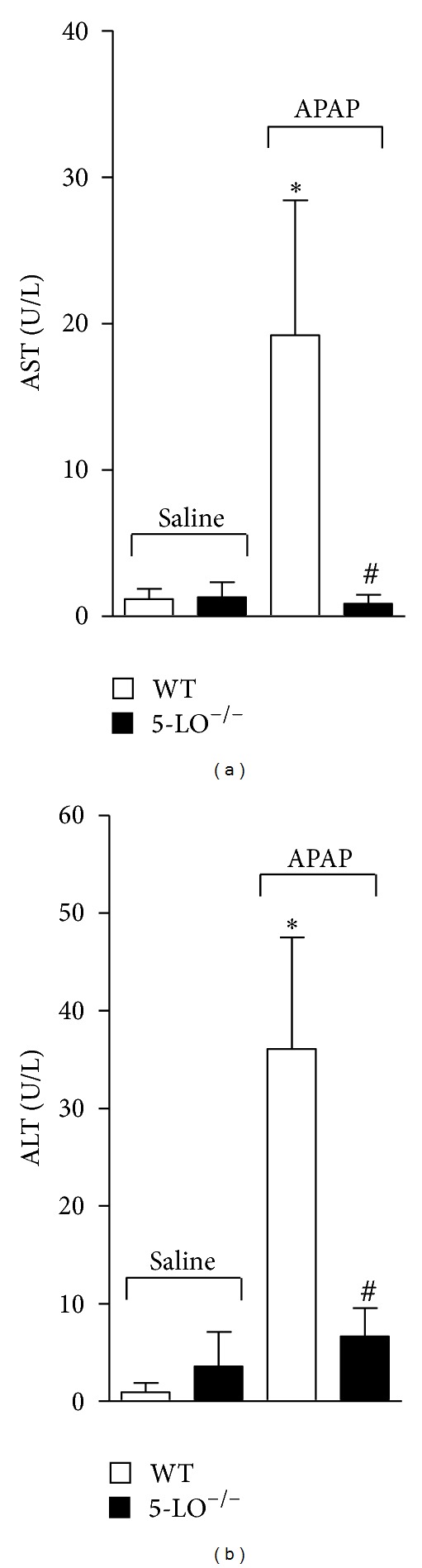
Acetaminophen (APAP) induces 5-LO-dependent liver damage. WT and 5-LO^−/−^ mice were treated with APAP (3 g/kg) or saline per oral, and after 12 h blood samples were collected to assess liver damage by measuring plasma levels of (a) aspartate aminotransferase (AST) and (b) alanine aminotransferase (ALT). Values are mean ± S.E.M., *n* = 5, representative of two separate experiments. *P < 0.05 compared to saline-treated WT and 5-LO^−/−^ mice and ^#^ *P* < 0.05 compared to APAP-treated WT mice. One-way ANOVA was followed by Bonferroni's multiple comparison test.

APAP (3 g/kg) or equal volume of saline per oral was administrated in 5-LO^−/−^ and WT mice, and after 12 h APAP-induced liver damage was estimated by plasmatic AST and ALT level determination. APAP significantly increased plasma levels of both enzymes in WT mice when compared to control group receiving saline but not in 5-LO^−/−^ mice (Figures 4(a) and 4(b)). There was no significant difference in AST and ALT levels between WT and 5-LO^−/−^ mice receiving saline. 

### 3.5. APAP-Induced Increase in MPO and NAG Activity Was Reduced in 5-LO^−/−^ Mice

**Figure 5:**
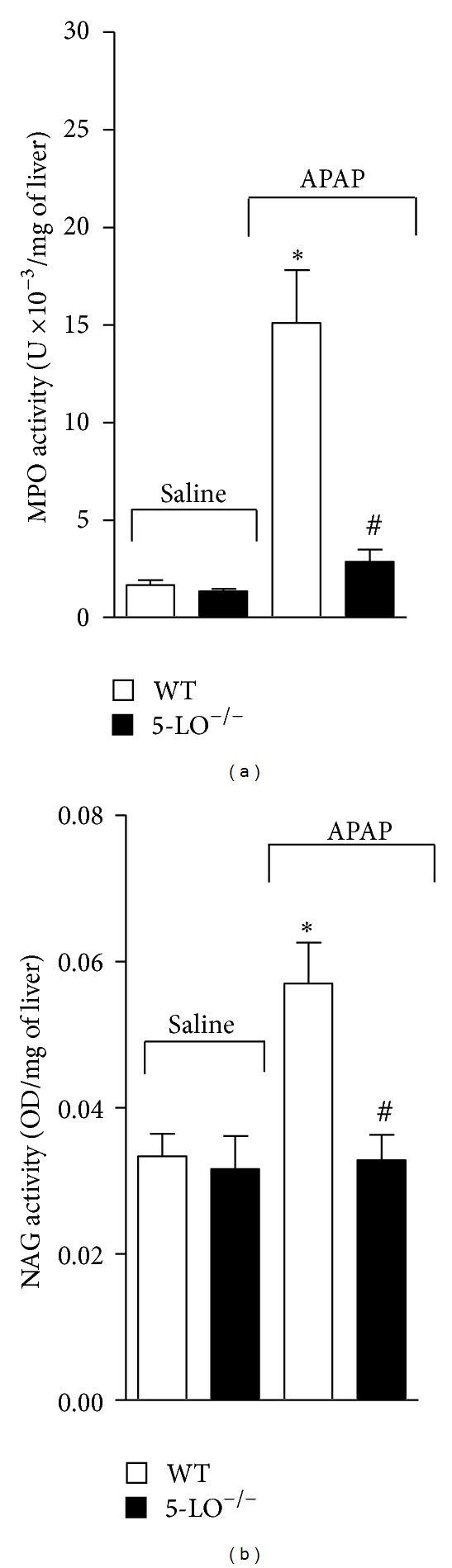
Acetaminophen (APAP) induces 5-LO-dependent neutrophil and macrophage recruitment. Neutrophil and macrophage recruitment to the liver was assessed by myeloperoxidase (MPO) and N-acetyl-*β*-D-glucosaminidase (NAG) activity determination in the liver 12 h after APAP (3 g/kg) or saline per oral treatment of WT and 5-LO^−/−^ mice. Values are mean ± S.E.M., *n* = 5, representative of two separate experiments. *P < 0.05 compared to saline-treated WT and 5-LO^−/−^ mice and ^#^ *P* < 0.05 compared to APAP-treated WT mice. One-way ANOVA was followed by Bonferroni's multiple comparison test.

The MPO and NAG activity were used as an indirect marker of neutrophil/macrophage and macrophage presence, respectively, in hepatic tissue 12 h after oral administration of APAP (3 g/kg) or equal volume of saline. APAP induced a significant increase of MPO and NAG activity in WT mice compared to saline (Figure 5). On the other hand, MPO and NAG activity were reduced in 5-LO^−/−^ mice compared to those in WT receiving APAP. No significant difference was found between MPO and NAG activity of WT and 5-LO^−/−^ mice that received saline. 

### 3.6. APAP-Induced Cytokine Production in the Liver Was Reduced in 5-LO^−/−^ Mice

**Figure 6:**
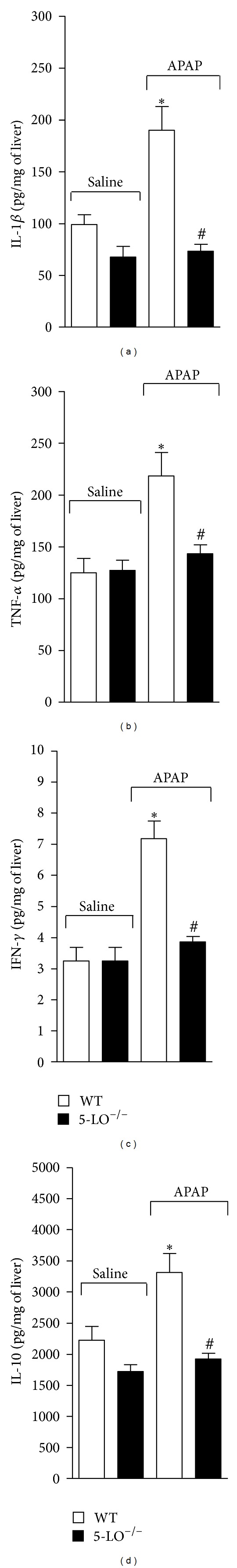
Acetaminophen (APAP) induces 5-LO-dependent induction of cytokine production in the liver. WT and 5-LO^−/−^ mice were treated with APAP (3 g/kg) or saline per oral, and after 12 h liver samples were collected to determine (a) IL-1*β*, (b) TNF-*α*, (c) IFN-*γ*, and (d) IL-10 levels by ELISA. Values are mean ± S.E.M., *n* = 5, representative of two separate experiments. *P < 0.05 compared to saline-treated WT and 5-LO^−/−^ mice and ^#^ *P* < 0.05 compared to APAP-treated WT mice. One-way ANOVA was followed by Bonferroni's multiple comparison test.

Mice were treated with APAP (3 g/kg) or equal volume of saline per oral, and after 12 h liver samples were collected and cytokine levels were determined. In WT mice, APAP induced significant increase of hepatic IL-1*β*, TNF-*α*, IFN-*γ*, and IL-10 production compared to saline (Figures 6(a), 6(b), 6(c), and 6(d), resp.). In 5-LO^−/−^ mice, however, APAP did not increase cytokine production. There was no significant difference in cytokine levels between WT and 5-LO^−/−^ that received saline. 

### 3.7. APAP-Induced Oxidative Stress in the Liver Was Reduced in 5-LO^−/−^ Mice

**Figure 7:**
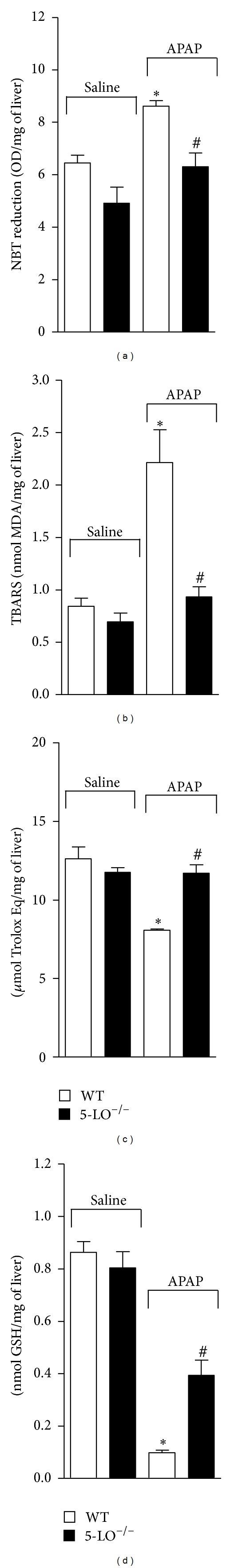
Acetaminophen (APAP) induces hepatic oxidative stress in a 5-LO-dependent manner. WT and 5-LO^−/−^ mice were treated with APAP (3 g/kg) or saline per oral, and after 12 h liver samples were collected to determine superoxide anion production (nitroblue tetrazolium (NBT) reduction) (a), lipid peroxidation (thiobarbituric acid reactive substances (TBARS)) levels (b), (c) reduced glutathione (GSH) levels, and (d) antioxidant capacity by 2,2′-azinobis(3-ethylbenzothiazoline 6-sulfonate; ABTS) assay. Values are mean ± S.E.M., *n* = 5, representative of two separate experiments. *P < 0.05 compared to saline-treated WT and 5-LO^−/−^ mice and ^#^ *P* < 0.05 compared to APAP-treated WT mice. One-way ANOVA was followed by Bonferroni's multiple comparison test.

Mice were treated with APAP (3 g/kg) or equal volume of saline per oral, and after 12 h liver samples were collected to determine superoxide anion production (NBT reduction), lipid peroxidation (TBARS levels), GSH levels, and antioxidant capacity by ABTS assay. WT mice treated with APAP presented significant increase of superoxide anion production (Figure 7(a)) and lipid peroxidation (Figure 7(b)) and decrease of GSH levels (Figure 7(c)) and antioxidant capacity (Figure 7(d)) compared to saline WT mice, which was not observed in 5-LO^−/−^ mice treated with APAP. There was no significant difference between WT and 5-LO^−/−^ that received saline. 

### 3.8. APAP-Induced gp91^phox^ mRNA Expression Was Reduced and Transcription Factor Nrf2 mRNA Expression Was Enhanced in 5-LO^−/−^ Mice

**Figure 8:**
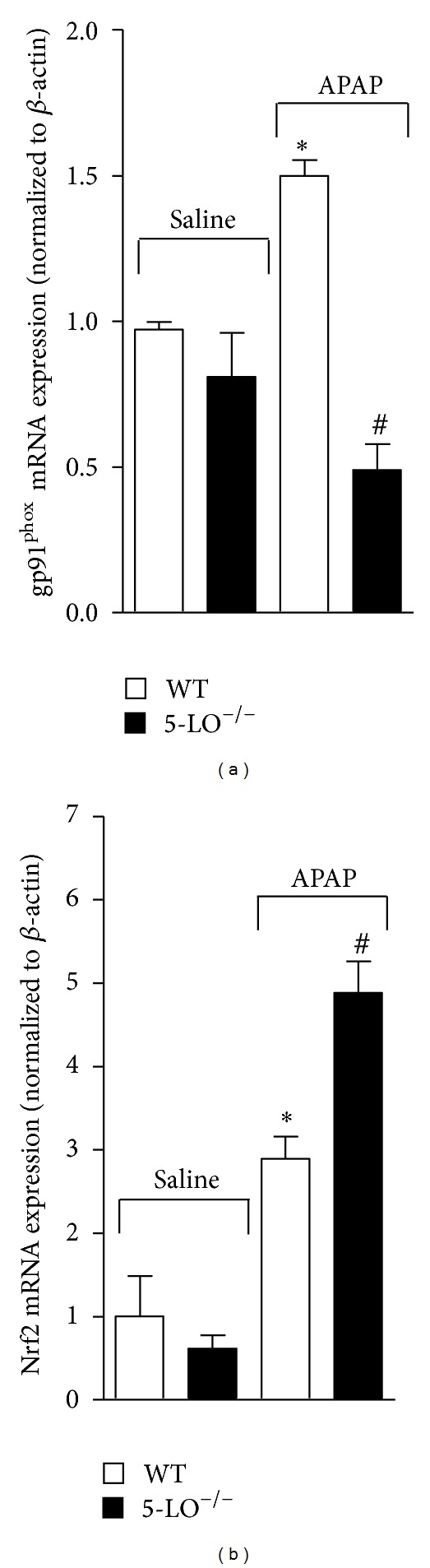
5-LO deficiency reduces acetaminophen (APAP)-induced increase of gp91^phox^ mRNA expression and increases transcription factor Nrf2 mRNA expression. The mRNA expression for gp91^phox^ (a) and Nrf2 (b) in the liver was assessed 12 h after APAP (3 g/kg) or saline per oral treatment of WT and 5-LO^−/−^ mice. Values are mean ± S.E.M., *n* = 4, representative of two separate experiments. *P < 0.05 compared to saline-treated WT and 5-LO^−/−^ mice and ^#^ *P* < 0.05 compared to APAP-treated WT mice. One-way ANOVA was followed by Bonferroni's multiple comparison test.

Mice were treated with APAP (3 g/kg) or equal volume of saline per oral, and after 12 h liver samples were collected to determine gp91^phox^ and Nrf2 mRNA expression by qPCR. WT mice treated with APAP presented significant increase of gp91^phox^ mRNA expression (Figure 8(a)) compared to saline, which was not observed in 5-LO^−/−^ mice treated with APAP. On the other hand, APAP-induced Nrf2 mRNA expression was enhanced in 5-LO^−/−^ mice compared to WT mice treated with APAP (Figure 8(b)). There was no significant difference between WT and 5-LO^−/−^ that received saline.

### 3.9. Pentobarbital-Induced Sleeping Time Was Similar in WT and 5-LO^−/−^ Mice

Mice were treated with sodium pentobarbital (50 mg/kg, intraperitoneal route), and sleeping time was assessed. WT (142.8 ± 10.10 min) and 5-LO^−/−^ (128.25 ± 7.25 min) mice did not present significant difference in pentobarbital-induced sleeping time (*P* = 0.363) (Table 1). Therefore, 5-LO^−/−^ mice did not present significant alteration in drug metabolism by CYP enzymes.

## 4. Discussion 

In most studies, hepatotoxicity is induced in mice by administrating 300–750 mg/kg of APAP [39, 40]; however, in this study a higher dose was used. Dose-response studies carried out in wild type (WT; Sv129) mice demonstrated that 3 g/kg of APAP is the submaximal lethal dose in this experimental model. The route of administration is certainly a contributing factor for this difference since in the present study APAP was administered per oral and not by intraperitoneal route [39, 40]. Another factor is that food restriction or fasting enhances susceptibility to APAP toxicity by CYP2E1 induction, enhancing ATP and GSH depletion [41, 42], which was not the case of the present study. Furthermore, the dose of APAP necessary to induce hepatotoxicity may also vary depending on mice strains. In Swiss mice, for example, 1.5 g/kg of APAP per oral induced a similar profile as 3 g/kg of APAP in Sv129 mice (data not shown).

APAP induced ~10-fold increase of LTB_4_ production in the liver. In agreement, 5-LO deficient (5-LO^−/−^) mice presented lower lethality rates compared to WT mice. The markedly higher lethality in WT mice lined up well with the higher degree of necrosis and liver damage in these mice, as assessed by liver histopathology analysis and plasma levels of AST and ALT. Furthermore, APAP-induced increase of MPO and NAG activity and cytokine production was reduced in 5-LO^−/−^ mice. APAP-induced oxidative stress was also reduced in 5-LO^−/−^ mice compared to WT mice, as observed by reduction of GSH depletion, lipid peroxidation, superoxide production, and increased total antioxidant capacity. Furthermore, there were reduced gp91^phox^ and increased Nrf2 mRNA expression in 5-LO^−/−^ mice compared to those in WT mice.

APAP induced 5-LO-dependent increase of the biochemical markers of neutrophils and macrophages, MPO and NAG activity. Excessive neutrophil and macrophage activity can contribute to perpetuation of inflammatory responses, additional liver damage, and even liver failure [25, 26] by releasing a series of proinflammatory molecules, such as cytokines [43], reactive oxygen species (ROS) [25], and proteases [44], that are responsible for further tissue damage and inflammation. Previous evidence [25, 26] together with the present results suggest that increased 5-LO-dependent neutrophil and macrophage recruitment/activity may contribute to liver damage induced by APAP. 

In the present study, APAP-induced IL-1*β*, TNF-*α*, IFN-*γ*, and IL-10 production in WT mice was reduced in 5-LO^−/−^ mice, suggesting that 5-LO products are involved in the production of cytokines induced by APAP. Cytokines are critical mediators of APAP hepatotoxicity. Previous studies report that the enhanced release of TNF-*α* and IL-1*β* may be responsible for further hepatic damage caused by NAPQI [45]. Interestingly, TNF*α* and IL-1*β* induce hepatic neutrophil and macrophage recruitment and activation [46, 47]. IFN-*γ* participates in APAP-induced liver injury by mediating leukocyte infiltration, hepatocyte apoptosis, and nitric oxide and cytokine (IL-1*α*, IL-1*β*, IL-6, and TNF-*α*) production [39]. Therefore, it is conceivable that these cytokines may contribute to the increase of neutrophil and macrophage markers in the liver, liver damage, and lethality in APAP intoxication. On the other hand, IL-10 is a potent anti-inflammatory cytokine capable of downregulating inflammation and is upregulated during severe liver damage as a protective mechanism against exacerbated tissue injury [48]. This is a possible explanation as to why increased IL-10 production was not observed in 5-LO^−/−^ mice after administration of APAP. 5-LO^−/−^ mice presented significantly reduced liver damage and inflammation when compared to WT mice; thus the endogenous upregulation of IL-10 was not observed. Furthermore, although it has been suggested that IL-10 may suppress proinflammatory cytokine production in the liver [49], in our study, IL-10 levels were not increased in 5-LO^−/−^ mice, suggesting that the reduction of IL-1*β*, TNF-*α*, and IFN-*γ* production observed in 5-LO^−/−^ mice was not dependent on the increased IL-10 production.

Another important finding of our study was that 5-LO deficiency improves antioxidant status in the liver of mice treated with APAP. APAP-induced increase of superoxide anion production (NBT assay) and lipid peroxidation (TBARS assay) and depletion of reduced glutathione (GSH) levels and overall oxidative buffering capacity of the liver (ABTS assay) of WT mice were prevented in 5-LO^−/−^ mice. Furthermore, a previous study reported that GSH levels correlate with ABTS profile as observed in the present study [50]. The production of superoxide anion by phagocytes such as macrophages and neutrophils is a crucial step in oxidative stress leading to lipid peroxidation and depletion of GSH and the overall endogenous antioxidant systems. In fact, APAP-induced increase of NADPH oxidase subunit, gp91^phox^, mRNA expression in the liver of WT mice was not observed in 5-LO^−/−^ mice. Furthermore, inflammation induces the expression of the transcription factor Nrf2, which is responsible for inducing the expression of antioxidant molecules including GSH [51]. In the present study, 5-LO deficiency resulted in an even greater expression of Nrf2 mRNA compared to that in WT mice, which further indicates an active role of 5-LO products during APAP intoxication to consume and limit antioxidant systems. In agreement, in acute lung injury mediated by oxidative stress and inflammation, inhibition of 5-LO by MK-886 significantly attenuated GSH depletion and lipid peroxidation in tissues [52]. Moreover, 5-LO deficiency inhibited leukocyte-derived ROS production and protected against degeneration of retinal capillaries in a mouse model of diabetic retinopathy [53]. This is consistent with the role that 5-LO plays in ROS generation by, for instance, activating NADPH oxidase resulting in superoxide anion production [54]. It is also important to consider the interactive system in which cytokines induce oxidative stress by stimulation of NADPH oxidase and ROS induce the activation of Nuclear Factor kappa B (NF*κ*B) and consequently cytokine production [24]. Therefore, it is possible that there is also an association between the inhibition of cytokine production and preservation of antioxidant systems observed in 5-LO^−/−^ mice.

The protection conferred by 5-LO deficiency in APAP-induced lethality was more evident in the first 12 h following APAP administration. Afterwards, although 5-LO^−/−^ mice presented less severe lethality when compared to WT mice, progressive lethality did occur. This might be related to the lack of lipoxin (LX) production in 5-LO^−/−^ mice since the synthesis of these important lipid mediators is dependent on 5-LO [55]. LXs present dual role in inhibiting inflammation and promoting resolution of the inflammation, which is essential for resolution of acute inflammatory processes and return to homeostasis [56]. Therefore, 5-LO inhibition seems to be more beneficial in the early stages of APAP intoxication when LT contribution to liver damage is critical. Moreover, the indirect inhibition of the 5-LO pathway may eventually be more beneficial in APAP intoxication since the inhibitor of 5-LO activating protein (FLAP), Bay-X-1005, significantly reduces LT biosynthesis and stimulated LX formation, resulting in further protection against CCl_4_-induced liver injury [9].

It is noteworthy that the sleeping time induced by pentobarbital was similar comparing WT and 5-LO^−/−^ mice. In agreement, 5-LO^−/−^ and WT mice do not present differences in liver CYP content and cytochrome c reductase activity [57]. Thus, the reduction of APAP-induced lethality and hepatotoxicity was not related to reduction of NAPQI formation by impaired activity of CYP.

In conclusion, the current study demonstrates that 5-LO participates in APAP-induced liver damage and lethality by enhancing LTB_4_ production in the liver. A lethal dose of APAP induced liver necrosis and inflammation, macrophage and neutrophil recruitment, cytokine production, and oxidative stress in the liver, all of which are reduced or abolished in 5-LO^−/−^ mice, therefore, elucidating the participation of 5-LO in these mechanisms of APAP hepatotoxicity. Furthermore, our findings suggest that inhibition of 5-LO may be a potential strategy to reduce the lethality and liver damage produced by APAP intoxication and, possibly, other types of liver damage that are mediated by similar mechanisms. Finally, although 5-LO deficiency did not abolish the lethality of APAP, it increased the survival rates following the ingestion of a lethal dose of APAP and prevented liver damage, which might add to the current therapeutic approaches to reduce APAP intoxication-induced death.

## Figures and Tables

**Table 1:** Effect of 5-LO deficiency on pentobarbital (50 mg/kg, i.p.)-induced sleeping time in mice.

Groups	Sleeping time (min)
WT	142.8 ± 10.10
5-LO^−/−^	128.25 ± 7.25

Data as mean ± S.E.M.; *n* = 10 per group, *P* = 0.363 versus WT (Student's *t*-test).
